# Asthma deaths in children in the UK the last straw!

**DOI:** 10.3399/bjgp24X738201

**Published:** 2024-04-30

**Authors:** Mark L Levy, Louise Fleming, Andrew Bush

**Affiliations:** London.; Consultant Respiratory Paediatrician, Imperial College Healthcare NHS Trust, London.; Professor of Paediatrics and Paediatric Respirology, National Heart and Lung Institute and Imperial Centre for Paediatrics and Child Health, Imperial College London; Royal Brompton Hospital, London.

The tragic death from asthma of a 10-year-old boy, William Gray, is a clear signal once again that doctors, nurses, and other healthcare professionals at all levels in the UK must take childhood asthma seriously. According to the coroner’s Regulation 28 report to prevent future deaths,^1^ William had a life-threatening asthma attack in October 2020. He was sent home by the accident and emergency (A&E) doctors only 4 hours after being brought in by the paramedics who had actually administered adrenaline on the way in. Maintenance inhaled corticosteroids (ICS) were not included on discharge from A&E. His GP then prescribed four courses of oral prednisolone for acute asthma attacks in the subsequent 7 months before he died on 29 May 2021. He was seen by a nurse practitioner on 25 May at the request of the GP following the fourth course of oral corticosteroids, but there was no escalation of his care or onward referral. He died 4 days later. The coroner concluded *‘that* [the courses of oral corticosteroids] *were insufficient to effectively manage obviously poorly controlled asthma in a picture of vastly excessive* [short-acting beta-2 agonist (SABA) bronchodilator] *reliever inhaler prescriptions and the absence ongoing of preventer medication.’*^1^ This coroner’s Regulation 28 report to prevent further deaths from asthma, published in December 2023, requires very urgent national action; it cannot be allowed to be forgotten, as have four previous Regulation 28s in which the lead author was expert witness. We are extremely saddened, and angry, that so many children continue to die from asthma in the UK and that action is not taken despite the same issues arising in the previous Regulation 28 reports and the Health Services Safety Investigations Body investigation in 2021.^2^

Many other UK doctors and nurses with expertise in asthma are extremely concerned about the ongoing poor asthma outcomes in the UK, particularly in children and young people (CYP). This is the most common chronic childhood disease and has relatively low priority on the national agenda. The UK has the highest number of asthma deaths in CYP in Europe^3^ and this is not decreasing.^4^

## Risk factors for asthma deaths known for six decades

We have known about risks for poor asthma outcomes for over six decades,^5^ and that excess use of reliever inhalers is life-threatening — this was made very clear in the National Review of Asthma Deaths (NRAD)^6^ and the 2019 Global Initiative on Asthma strategy document (updated annually: https://ginasthma.org). We have also known for nearly 30 years that insufficient ICS usage in asthma is associated with asthma attacks and deaths. Furthermore, the NRAD recommended that anyone having two asthma attacks in a year should be referred to an asthma specialist. Also, because we have known for many years that one life-threatening attack is the greatest risk factor for another, such patients should be followed up by an asthma expert long-term. Yet William Gray’s death in May 2021 was preceded by all four of these risk factors that were not acted on as mandated by published evidence and guidance.

The NRAD confidential enquiry that was commissioned by the four devolved nations demonstrated that more than two-thirds of those who died from asthma between February 2012 and January 2013 had major preventable features.^6^ We had data for 28 of the 36 CYP who died from asthma (missing eight, because the doctors failed, despite numerous requests, to send us the records). In total, 27/28 of these CYP were treated below an acceptable standard according to the expert panels participating in the enquiry. The NRAD, published the day before World Asthma Day, 5 May 2014, made 19 recommendations based on 17 key findings, which to date have not been implemented in the UK.^6^

We previously described three other potentially preventable CYP asthma deaths.^7^ These children, aged 9, 10, and 14 years, had multiple asthma attacks, most of which were not clearly coded in the general practice records, and all three had been prescribed excess SABA reliever inhalers. Yet none of these children were recognised as being at risk, and none were referred by their GPs or hospital doctors to asthma specialists.

Sadly, asthma deaths and Regulation 28 statements only result in short-lived publicity and, to our knowledge, only one^8^, after many years of campaigning by her mother, resulted in government action regarding air pollution, after the coroner concluded that air pollution was responsible for Ella Kissi-Debrah’s death due to asthma.^9^

## Major preventable risk factors keep recurring

The potentially preventable factors in the NRAD and in the cases investigated in inquests keep recurring. Asthma attacks are all too often treated as if they were a single acute episode with no long-term consequences. There is a total failure to recognise the risks posed by repeated acute episodes. Acute asthma is treated as if it is a short-lived inconvenience, rather than a red flag that an ongoing chronic disease is out of control.^5^

In our view (and that of others) asthma is just not taken seriously enough in the UK. It is a very common disease; depending on how it is defined, at least 9.6% of the UK population have asthma. The disease accounts annually for at least 6.3 million primary care consultations, 93 000 hospital in-patient episodes, 1800 intensive-care unit episodes, and 36 800 disability living allowance claims.^10^ According to the charity, Asthma and Lung UK, asthma kills three people every day, and every 10 seconds someone has a potentially life-threatening attack.^11^ Yet, asthma is just not high up on the national health agenda. Health services in the UK are under-resourced for caring for this population: the UK has the highest number of severe asthma hospitalisations and the second lowest number of adult and paediatric respiratory specialists per 100 000 in 29 OECD countries.^12^

## Asthma is more than an acute disease

Because asthma is treated as if it is a series of acute isolated episodes, rather than a long-term chronic disease, too often attacks are trivialised. They are managed without formal follow-up or referral to specialists, and worryingly much of the asthma care is delegated to individuals without appropriate training or expertise — which contravenes the newly emphasised requirement by the UK General Medical Council that doctors should only delegate care to appropriately trained individuals.^13^ Of course we understand how hard our colleagues are working and how resources in the NHS are scarce; however, given that asthma is the commonest chronic childhood disease, in our view all doctors and nurses caring for these young people should be familiar with current evidence-based treatment of asthma and possess the appropriate knowledge and skills as set out in the National Capabilities Framework for professionals who care for CYP with asthma.^14^

We have reached a point in the UK where urgent and immediate national action is needed to stop asthma attacks and deaths in children. A primary care audit reduced hospital admissions by 16%.^15^ Using similar methodology, a 7-step action plan for GP practices^16^ may help to raise awareness of the risk factors, change practice, and reduce workload, attacks, and deaths. The plan includes systematic critical review of all asthma attacks, identification and dealing with modifiable risk factors, and improvement of practice systems.

These attacks are preventable in almost all cases with appropriate medical management and education. Above all we must recognise that an asthma attack is a red-flag, never event that merits a detailed and focused response, and not a mere box-ticking exercise by whoever happens to be holding a pencil at the time.
